# Adipophilin as prognostic biomarker in clear cell renal cell carcinoma

**DOI:** 10.18632/oncotarget.15639

**Published:** 2017-02-23

**Authors:** Yuri Tolkach, Christine Lüders, Sebastian Meller, Klaus Jung, Carsten Stephan, Glen Kristiansen

**Affiliations:** ^1^ Institute for Pathology, University Hospital Bonn, Bonn, Germany; ^2^ Berlin Institute of Urologic Research, Berlin, Germany; ^3^ Department of Urology, Charité, Universitätsmedizin Berlin, Berlin, Germany

**Keywords:** renal cell carcinoma, clear cell, adipophilin, perilipin 2, PLIN2

## Abstract

**Objective:**

To study the expression of adipophilin (*PLIN2*), a lipid storage-associated cell protein, in different subtypes of renal cell cancer and to elucidate its prognostic value.

**Materials and Methods:**

Two-hundred-seventy-five patients with renal cell carcinoma (RCC) were included in this study. Immunohistochemistry with a polyclonal antibody to adipophilin was used on the tissue microarray (formalin-fixed, paraffin-embedded tissue) for detection of adipophilin. Median follow-up time was 91 (range 1-159) months in the whole cohort and 100 (1-159) months for patients with clear-cell RCC. Additional validation for adipophilin was performed using publicly available gene expression data for clear cell RCC from The Cancer Genome Atlas (TCGA).

**Results:**

Adipophilin expression was detected in 14.3% of papillary RCC, in 0% of chromophobe RCC and in 58.7% of clear-cell RCC in the cytoplasm or at the membrane. Only membrane expression was correlated with other clinical parameters (pT-stage, pN-stage, R-status, sex) and showed a prognostic significance in univariate analysis with regard to overall survival of patients with clear cell subtype (HR 2.90, 95% CI 1.55-5.42, p=0.001), which failed significance on multivariate analysis. mRNA expression of *PLIN2* on TCGA data using best selected cut-off was prognostically significant in both univariate (HR 1.76, 95% CI 1.28-2.42, p = 0.0005) and multivariate analyses (HR 1.46, 95% CI 1.05-2.04, p = 0.0257).

**Conclusions:**

Adipophilin is a novel and still understudied prognostic biomarker in clear cell renal cell carcinoma which deserves further study.

## INTRODUCTION

Kidney cancer is one of the ten most frequently occurring cancers with substantial mortality estimates in Western societies [1]. Constant search for powerful biomarkers capable of prognostication and prediction of therapy response in this type of tumors is one of the priorities of modern medicine and science.

Importantly, all tumors are considered to undergo certain metabolic changes to achieve high proliferation rates and sustain further growth. At that, lipid metabolism supposed to be seriously altered in many tumor types [2, 3]. Adipophilin is one of the important players in lipid metabolism, responsible for storage of lipid droplets in all types of cells [4, 5]. Some recent studies show that many tumors overexpress adipophilin [6–22], especially those with clear cell histology [10, 11]. Several small reports showed that renal cell carcinoma (RCC) cells also overexpress adipophilin [10, 11, 23–27] and one major study [24] reported the prognostic role of this biomarker in clear cell subtype on transcript level. However, an analysis on the protein level was pending, so far.

In this study we aimed to investigate the expression patterns of adipophilin using immunohistochemistry in patients with different subtypes of the renal cell cancer and to study the prognostic effect of this biomarker on the survival of patients Table 1. For further validation we have used the publicly available expression data set of *PLIN2* (adipophilin) from The Cancer Genome Atlas (TCGA).

**Table 1:** Clinical characteristics of patient cohort with RCC (n=275)

Parameter	Absolute	Proportion
Age, years mean (range)	61.0 (30-86)	-
Follow-up, months median (range)	91.0 (1-159)	-
Status to the end of follow-up		
alive	192	69.8%
dead	83	30.2%
Sex:		
male	187	68.0%
female	88	32.0%
Histological subtype:		
clear cell	230	83.6%
papillary	35	12.7%
chromophobe	10	3.7%
pT-Stage (TNM 2002):		
pT1a	100	36.4%
pT1b	66	24.0%
pT2	21	7.6%
pT3a	32	11.6%
pT3b	50	18.2%
pT3c	4	1.5%
pT4	2	0.7%
WHO/ISUP grade:		
1	48	17.5%
2	154	56.0%
3	47	17.1%
4	16	5.8%
N/A^a^	10	3.6%
pN-Stage:		
N0	146	53.1%
N+	16	5.8%
Nx	113	41.1%
Lymphadenectomy		
Yes	150	54.4%
No	125	45.6%
M-Stage (clinical)		
M0	245	89.1%
M1 synchronic	19	6.9%
M1 asynchronic	11	4.0%
R-status		
R0	250	90.9%
R1	10	3.6%
R2	4	1.5%
not available	11	4.0%
Adrenalectomy		
Yes	174	63.3%
No	101	36.7%
ECOG performance status		
0	189	68.8%
1	79	28.7%
2	7	2.5%

Comments: a - No grading for chromophobe subtype

## RESULTS

### Immunohistochemistry: staining patterns

**Figure 1:**
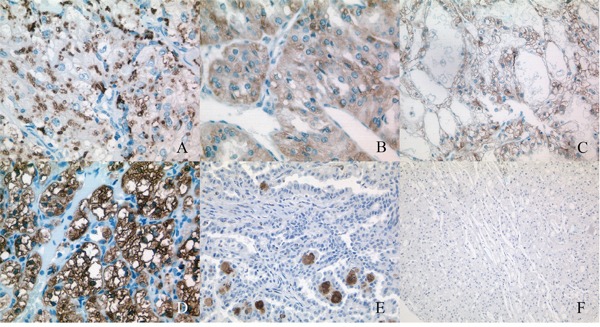
Staining patterns of the adipophilin in renal cell carcinoma (RCC) **(A)** Vesicular (drop-like) staining of cytoplasm. **(B)** Vesicular and granular staining of cytoplasm. **(C)** Weak membrane staining. **(D)** Strong membrane staining and vesicular and granular staining of cytoplasm. **(E)** No staining of tumor cells in papillary RCC, granular staining of cytoplasm of macrophages (foamy cells), could be mistaken as tumor cells. **(F)** No staining in chromophobe RCC.

Adipophilin immunohistochemistry shows immunoreactivity of cytoplasm, membrane or both (Figure 1). Cytoplasm staining of RCC tumor cells showed vesicular (drop-like) or granular pattern. Cell nuclei were negative in all cases. There was no noteworthy heterogeneity of immunoreactivity at the level of individual cells. The frequency and the expression levels are displayed in Table 2. Since immunoreactivity to adipophilin was almost exclusively detected in clear cell RCC (CC-RCC), we restricted the further analysis to these 230 patients (Supplementary Table 1 for clinical characteristics). Among these, 95 patients were completely negative, 135 patients were positive at one (membrane or cytoplasm) or both locations at any staining grade.

**Table 2:** Results of immunostaining using adipophilin antibodies in all subtypes of renal cell carcinoma (n=275)

Staining	Membrane^a^		Cytoplasm^b^	
	frequency	percentage	frequency	percentage
0	Negative	155	56.3%	207	75.3%
1	Weak	45	16.4%	56	20.3%
2	Moderate	69	25.1%	12	4.4%
3	Strong	6	2.2%	0	0.0%

Comments: ^a^ all positive cases are clear cell subtype, except 2 case of papillary subtype (1x weak and 1x moderate staining), ^b^ all positive cases are clear cell subtype, with except of 3 other cases with papillary subtype (3x weak)

### Associations between adipophilin expression and clinical parameters

**Figure 2:**
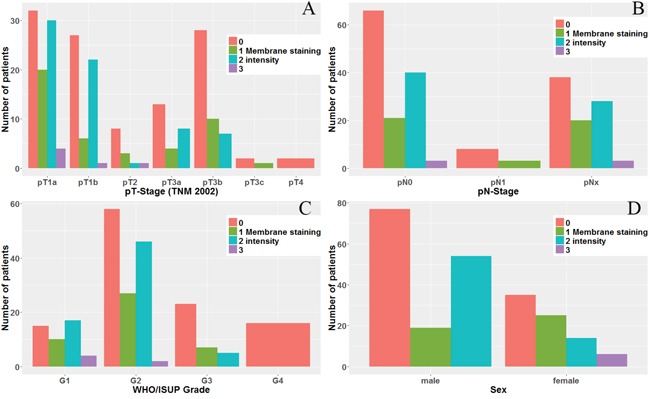
Membrane staining patterns of adipophilin in clear cell RCC with regard to: **(A)** pT-stage (p=0.001), **(B)** pN-stage (p=0.096, in pN1 number of cases < 10), **(C)** WHO/ISUP Tumor grade (p<0.0001), and **(D)** Sex of the patient (p<0.00001).

Cytoplasm positivity for adipophilin demonstrated no significant associations with common prognostic parameters (staging, grading) and other important patient-related variables (sex, age). Quite the contrary, membrane staining was more intensive in patients with less advanced and higher differentiated tumors. Furthermore women compared to men presented with membrane positivity more often (Exact Fischer's test p<0.00001; Figure 2). At that, all six cases of strong (“3”) membrane immunoreactivity were female patients. No associations were found for patient age.

### Survival analyses

**Figure 3:**
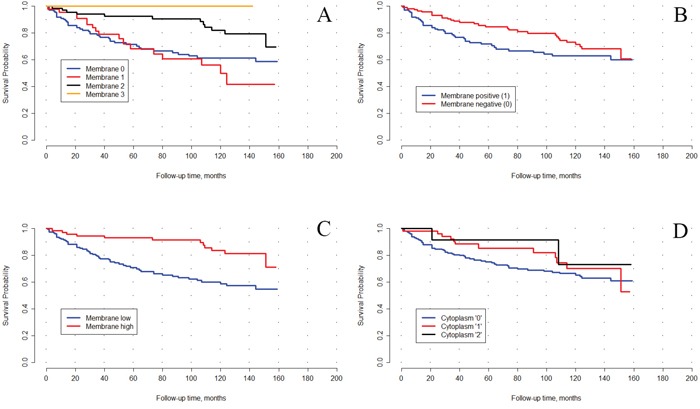
Kaplan–Meyer curves for overall survival of patients with clear cell RCC after tumor excision, stratified based on the adipophilin immunostaining (p-level using long-rank): **(A)** membrane staining (4-tier), p=0.014, **(B)** membrane staining (positive/negative), p=0.368, **(C)** membrane staining high (moderate and strong) vs low (negative and weak), p=0.006, **(D)** cytoplasm staining, 3-tier (negative, weak or moderate; there were no cases with strong cytoplasm staining), p=0.721.

Kaplan Meier analysis with log-rank test (Figure 3), as well as univariate analysis (Cox regression) (Table 3) demonstrated that only membrane staining of adipophilin was prognostically relevant for overall survival (in univariate analysis HR 2.90 for low vs high membrane expression for negative outcome, 95% CI 1.55-5.42, p=0.001). However, on multivariate analysis adipophilin expression at the membrane showed no independent prognostic value (Table 4).

**Table 3:** Univariate analysis of prognostic significance of different factors for overall survival in patients with clear cell renal cell carcinoma, n=230 (Cox regression)

N	Parameter	HR	95% Confidence interval	Significance
1	pT-stage			
	pT1	1	-	-
	pT2	2.68	1.01-7.12	0.048
	pT3a	3.61	1.74-7.49	0.001
	pT3b-pT4	7.05	4.05-12.29	<0.0001
2	pN-stage: pN1 vs pN0/pNx	6.1	2.97-12.53	<0.0001
3	R-status, pos. vs neg.	9.41	4.56-19.44	<0.0001
4	WHO/ISUP grade			
	1	1	-	-
	2	3.0	1.18-7.64	0.021
	3	4.22	1.52-11.73	0.006
	4	16.07	5.62-45.9	<0.0001
5	Age, continuous	1.02	1.00-1.05	0.123
6	Sex, female vs male	0.87	0.52-1.43	0.572
7	ECOG status			
	0	1	-	-
	1	3.53	2.16-5.77	<0.0001
	2^a^	17.76	6.66-47.32	<0.0001
8	Membrane, 4-tier			
	0	1	-	-
	1	1.18	0.67-2.08	0.572
	2	0.4	0.21-0.76	0.005
	3	<0.001	-^b^	0.963
9	Membrane, low vs high	2.90	1.55-5.42	0.001
10	Membrane, positive vs negative	0.66	0.41-1.06	0.085
11	Cytoplasm, 3-tier			
	0	1	-	-
	1	0.69	0.37-1.29	0.691
	2	0.57	0.13-2.12	0.359

Comments: ^a^ - < 10 patients, ^b^ - {<0.0001 – 1.23E+231}, 6 outlier cases in analysis

**Table 4:** Multivariate analysis of prognostic significance of selected factors for overall survival in patients with clear cell renal cell carcinoma, n=230 (Cox regression)

N	Parameter	HR	95% Confidence interval	Significance
1	pT-stage			
	pT1	1	-	-
	pT2	1.34	0.44-4.06	0.603
	pT3a	2.40	1.08-5.33	0.032
	pT3b-pT4	4.27	2.27-8.05	<0.0001
2	pN-stage			
	pN0/pNx	1	-	-
	pN1*	0.74	0.28-1.91	0.531
3	R-status			
	R0	1	-	-
	R+	1.77	0.66-4.78	0.259
4	WHO/ISUP grade			
	1	1	-	-
	2	2.59	1.00-6.72	0.051
	3	2.15	0.73-6.36	0.166
	4	7.53	2.44-23.25	<0.0001
5	ECOG status			
	0	1	-	-
	1	2.38	1.35-4.21	0.003
	2^a^	5.78	1.38-24.17	0.016
6	Membrane staining			
	high	1	-	-
	low	1.52	0.76-3.02	0.235

Comments: ^a^ - less than 10 cases

### Analysis of TCGA cohort

**Figure 4:**
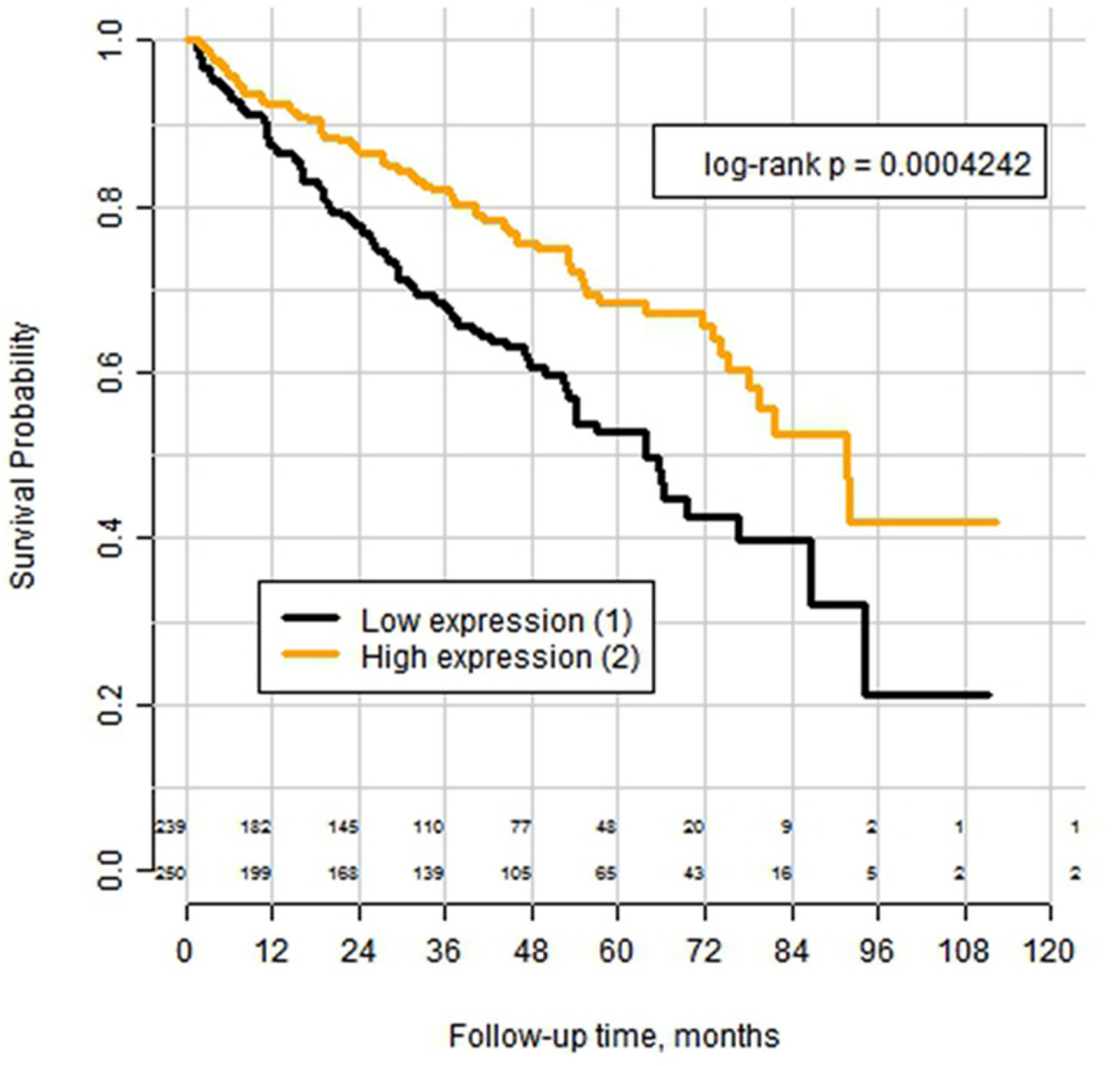
Kaplan–Meyer curves for overall survival of patients with clear cell RCC from the TCGA-cohort (n=489) after tumor excision, stratified according the mRNA expression of the PLIN2 gene (adipophilin) with the expression cut-off = 17995 transcript calls

The mean expression of *PLIN2* mRNA in tumor tissue was 22678 (range 279-159128), in normal renal parenchyma 2855 (range 911-25707) with significant difference (p<0.0001). The best cutoff according to the Cox-model-based selection was 17995, splitting the cohort into subgroups with low (n=239) and high (n=250) expression of *PLIN2* mRNA (See Supplementary Table 4). Log-rank analysis of the Kaplan–Meyer curves (Figure 4) revealed significantly better overall survival in the subgroup with high expression (p=0.0004). Univariate Cox analysis HR for this cut-off showed the HR of 1.76 (95% CI 1.28-2.42) with p = 0.0005 (See Supplementary Table 2). In multivariate Cox analysis with inclusion of pT-stage, pN-stage and WHO/ISUP-Grade of the tumor into the model the HR for low expression vs high expression was 1.46 (95% CI 1.05-2.04) with p = 0.0257 (see Supplementary Table 3). When *PLIN2* mRNA-expression was used as continuous parameter, the significance level was of the same order: in univariate Cox analysis HR was 0,986 (0,976-0.996) for step-wise increase at every 1000 transcripts of mRNA (p=0,005), in multivariate analysis using the same set of the parameters as for cut-off-based version of model – HR 0,989 (0,979-0,999) for step-wise increase at every 1000 transcripts of mRNA (p=0,045).

## DISCUSSION

Adipophilin (adipocyte differentiation-related protein, perilipin-2) is a member of the perilipin protein family, consisting of 3 members (also perilipin-1 and perilipin-3), which coat the lipid droplets in the cytoplasm of diverse cells and therefore allow the storage of energy-rich fats [4]. The functional correlations are almost mechanistic: accumulation of lipids in the cell will lead to increased expression of adipophilin. This process could be indeed observed in its purity in adipocytes, where adipophilin was first described as a membrane-associated protein [5]. Although the function of adipophilin as lipid droplet wall component is relatively straightforward, many authors consider, that its functions extend far beyond the structural role. It was shown, that modulation of adipophilin expression in hepatocytes in mice affected expression of 1363 genes with primary metabolic orientation [29].

Adipophilin is not a tumor-specific biomarker, as it was shown, that adipophilin expression is affected in a wide range of metabolic disorders in almost all tissue types [4]. Nevertheless, many tumor types are recognized for their lipid metabolism disturbances, which supposed to be partly related to the metabolic reprogramming of the tumor cells and issues, related to energy consumption in a hypoxic microenvironment [2, 3]. Thus, adipophilin fell into the scope of cancer researchers and was already addressed in several tumor types as being overexpressed [6–22]. Two studies showed that most tumors with clear-cell histology accumulate adipophilin [10, 11]. Moreover, at the level of mRNA expression *PLIN2* (gene coding adipophilin) appears to be an outlier for virtually all tumor entities as summarized using cancer outlier profile analysis in the Oncomine database (see Supplementary Discussion 1).

In our study only 5/35 cases of papillary RCC tumors were positive to adipophilin using immunohistochemistry. All 10 chromophobe tumors were negative. Positive staining at one of two locations (membrane or cytoplasm; nuclear staining was not observed) was detected in 58.7% (135/230) of patients with CC-RCC with higher expression in less aggressive tumors. This is in trend of previous studies.

Two studies from one group [23, 24] revealed that high *PLIN2* mRNA expression is associated with a favorable cancer-specific survival in CC-RCC patients. Metastatic lesions uniformly showed low expression. In these two [23, 24] and several other studies [11, 26, 27] expression in CC-RCC was uniformly several times higher than in normal tissue, papillary and chromophobe RCC with expression in papillary subtype significantly higher than in chromophobe RCC. Overall rate of overexpression of adipophilin in CC-RCC cells was in a wide range from 64% up to 100% [10, 24, 30]. Therefore it could be stated, that expression estimation at the mRNA level [24] and protein level (our data) are well concordant. Adipophilin was also a promising diagnostic marker of RCC in urine (for both CC-RCC and papillary RCC; no effect for chromophobe RCC, bladder cancer, prostate cancer, benign oncocytomas, angiomyolipomas) [25].

However, using mRNA-based techniques for biomarker detection in routine practice is challenging due to degradation issues. Our study represents the first extensive evaluation of the adipophilin expression in renal cell carcinoma at the protein level with regard to its prognostic significance. Importantly, only membrane positivity in our study was correlated with clinical parameters (tumor stage, grade, pN-status) and showed a prognostic value for overall survival in Kaplan–Meyer and univariate Cox-regression analysis. Localization of adipophilin at the cell membrane is not fully clear from the molecular biological point of view, although some studies provide the explanation for this phenomenon in terms of lipid droplet formation machinery in the cell membrane in certain circumstances and of the role of adipophilin in active transport through cell membrane. Some other technical reasons for this could be also mentioned (See Supplementary Discussion 2).

Yao et al. [24] demonstrated a multivariate prognostic significance of adipophilin on transcript level, which we could confirm in a validation analysis using publicly available mRNA expression data from The Cancer Genome Atlas. However, in our attempt to validate this further on protein level, we failed, as membranous adipophilin lost its significance on multivariate analysis.

The functional role of adipophilin in tumor cells is yet unclear. Overexpression of *PLIN2* was shown to lead to lipid overload [32, 33, 34]. In RCCs specifically, *PLIN2* overexpression may be induced by VHL mutations [23]. The induction of the *PLIN2* expression is also possible through the hypoxia [35–37], which is a cornerstone of VHL pathway alterations in CC-RCC [38]. This could explain the lipid overload in this type of tumor. The presence of VHL mutation in tumor cells has been shown to carry a survival benefit for patients with CC-RCC, which could explain the less aggressive nature of the tumors with overexpressed adipophilin ([23, 24] and our study). Two further interesting studies [39, 40] point at cytoprotective overexpression of *PLIN2* as the defense mechanism of the cell to lipid overload, which would otherwise lead to the apoptosis due to lipid toxicity given the improper storage of lipids in the cytoplasm [40]. There is also data showing that accumulation of the lipid droplets could have a malignancy promoting effect in tumors (See Supplementary Discussion 3) [9, 41]. This seems not to be a proper explanation of adipophilin overexpression in RCC.

One other interesting finding in our study is the differences in adipophilin expression in CC-RCC cases between male and female patients. Given the fact, that higher expression supposes less aggressiveness and taking into account the overall male to female ratio in RCC with approximately 2-3:1 [1], one could speculate, that in women tumor biology could be different. Earlier such correlations were never showed for adipophilin expression in RCC and other tumor types [11, 24], although only one study [24] embraced enough cases to reveal any associations with sex in RCC. Increased expression of *PLIN2* in women was earlier showed in skeletal muscle in one study [42], underpinning some physiological differences in lipid metabolism of different sexes.

One of the potential limitations of our study could be the undersampling issue. It is well known that some tumors (including renal cell carcinoma) show substantial intratumoral heterogeneity, the extent of which is still understudied [43]. We have analyzed two tumor samples per patient and noticed significant differences of adipophilin expression only in a single patient of our cohort, which endorses the assumption of a relatively homogenous expression of adipophillin in renal cancer.

Drawing a conclusion, adipophilin seems to play an important role in metabolism of RCC and, probably, of many other tumor types (especially with clear-cell phenotype). Even though we could not find a multivariate prognostic value in our data set, which could be also related to some limitations (particularly the number of patients), the prognostic role of adipophilin expression in CC-RCC is robustly corroborated by the analysis of TCGA. Further studies on adipophilin expression are warranted with the use of different antibodies and correlations between mRNA expression and immunohistochemistry.

This study demonstrated, that adipophilin expression is correlated with many important clinical parameters in patients with clear cell RCC and has a univariate prognostic value in two independent cohorts of CC-RCC. Further studies are needed to clarify if the multivariate prognostic value observed on the transcript level can be verified on the protein level with protocol modifications. Also, the relationship of adipophilin expression and metabolic alterations in tumor cells warrants additional investigations, preferably using integrative methodologies (parallel mRNA and protein levels).

## MATERIALS AND METHODS

### Patient selection

We have included 275 patients, who were operated on in the urological department of Charité Hospital (Berlin, Germany) due to renal cancer between 1993 and 2004. Final pathology confirmed that all patients had a RCC of different histological subtypes. Cases were selected according to tissue availability, without using any further selection criteria. Patients with accompanying secondary malignancies were initially excluded. All patients were prospectively followed-up. The clinical characteristics of the patient cohort are presented in the Table 1. For this cohort we had the data available on overall survival with median follow-up exceeding 7,5 years.

### Materials

For our study we have used the formalin-fixed paraffin embedded archive tissue. Initially tumor and normal tissues were fixed in the neutral buffered 10% formalin according to the institutional standards. For all 275 patients tumor and normal tissue were representatively available.

### Construction of tissue microarray (TMA)

A tissue microarray was constructed as described in [28]. Briefly, for every patient two cores of tumor and adjacent normal tissue were taken, each 1 mm in diameter, which were compiled in five paraffin blocks. Histological control of the tissue content was carried out by an experienced uropathologist.

### Immunohistochemistry protocol

The tissue microarray was cut (3 μm) and mounted on superfrost slides (Menzel Gläser, Brunswick, Germany). After deparaffinization with xylene and gradual rehydration, antigen retrieval was achieved by pressure cooking in 0.01 mol/L citrate buffer for 5 min. Slides were incubated with primary antibody (rabbit polyclonal adipophilin antibody; Cell Marque (Rocklin, California, USA); 393-A16; dilution 1:25). The slides were counterstained with hematoxylin and aqueously mounted.

### Immunohistochemistry evaluation

The immunohistochemic staining was evaluated by two experienced pathologists, blinded for clinical outcome. The staining intensity was evaluated using a 4-tier grading system (0: negative; 1: weakly positive; 2: moderately positive; 3: strongly positive) for membrane and cytoplasm separately. For further statistical analysis the moderately and strong adipophilin positive tumors (Staining grades 2 and 3) were grouped against the weakly stained and negative ones (Staining grades 0 and 1). Statistical analysis was performed for membrane and cytoplasm stainings separately and also using a pooled analysis.

### Ethical issues

Permission of the institutional ethical committee (Charité, Berlin, Germany) was received for this study.

### Analysis of the TCGA data

The extraction of the TCGA data (clinical data, mRNA expression data) was carried out for clear-cell subtype of the RCC (data version 28.01.2016). These data were available for 533 patients with expression data generated using the Illumina HiSeq 2000 RNA Sequencing platform (Version 2). Normalized results of the mRNA expression were used for analysis. Overall survival was used as the end-point. mRNA expression for *PLIN2* was extracted for every patient/tumor, as well as for normal kidney tissue, available for 73 patients. Some modifications of the database were performed before the analysis: exclusion of the patients with internally (by TCGA committee) revealed discrepancies in the clinical information, exclusion of the patients with follow-up < 30 days and with incomplete clinical information. Given these modifications 489 patient cases were available for analysis with 158 events (death) during follow-up in this group. Two types of multivariate analyses were carried out with regard to tumor grade (1. All patients; 2. The patients with tumor grades G2, G3 and G4), while there are only few patients (n=8) with G1 tumors in the modified TCGA cohort, which uniformly demonstrate excellent survival statistics, which distorts the Cox statistics.

### Statistical analysis

Statistical analysis was done using SPSS, version 22.0 (IBM Corporation, Armonk, New York, U.S.) and R (version 3.2.2). Kaplan–Meyer curves, log rank test, univariate and multivariate Cox proportional hazards regression analysis were used for analysis of prognostic significance of adipophilin with regard to other clinical prognostic parameters. Extraction of the TCGA mRNA expression data was done in R using TCGABiolinks package. The best cut-off for the mRNA expression on the TCGA data was selected in R using the survMisc package (Cox-based selection).

## SUPPLEMENTARY MATERIALS FIGURES AND TABLES





## Abbreviations

CC-RCC: clear cell renal cell carcinoma
HR: hazard ratio
ISUP: Internation Society of Urological Pathology
RCC: renal cell carcinoma
TCGA: The Cancer Genome Atlas
WHO: World Health Organization
