# Supplementary material for: Adipophilin as prognostic biomarker in clear cell renal cell carcinoma
Source: Oncotarget. 2017 Feb 23;8(17):28672–82. doi: 10.18632/oncotarget.15639 (PMC5438682; doi:10.18632/oncotarget.15639)
Supplement: Supplementary file 1 [file oncotarget-08-28672-s001.pdf]

Adipophilin as prognostic biomarker in clear cell renal cell carcinoma

SUPPLEMENTARY DATA

At the level of mRNA expression *PLIN2* (gene coding adipophilin) appears to be an outlier for virtually all tumor entities as summarized using cancer outlier profile analysis in Oncomine [1] (in 191 of 970 cancer datasets *PLIN2* is among top 10% of outlier genes, both over- and underexpression). The tumor types with *PLIN2* being an outlier in more than 10 datasets are kidney cancer, breast cancer, leukemia and prostate cancer. The clinical significance of these impressing findings was never studied in details and confirms once more time the importance of metabolic changes in tumor cells.

Localization of adipophilin at the cell membrane is not fully clear from the molecular biological point of view, although some studies provide the explanation for this phenomenon. Given the function of this protein it is awaited to be detected in the cytoplasm in a vesicular/granular (drop-like) pattern which would correspond to lipid droplets being preserved in cell cytoplasm. Solely cytoplasmic drop-like pattern on immunohistochemistry was earlier reported in RCC [2, 3] and other tumors. Membrane staining facing the basement membrane side of the tumor cells was, so far, described only for colonic adenocarcinomas [4].

Immunostaining patterns of adipophilin could stem from its biological functions. Adipophilin has high affinity to neutral lipids and phospholipids of LD wall, protecting the first from lipolysis and facilitating the storage lipids being the main function of this protein [5, 6]. Free adipophilin not succeeded to connect to the lipids is being rapidly cleared via ubiquinitation [7, 8]. Therefore, the immunogenicity of adipophilin could be substantially reduced in a “stabilized” state. Lipolysis promotes the release of the PLIN2 from the connections with lipids making it detectable in cytoplasm [7, 8], therefore affecting the immunohistochemistry-based detection in cytoplasm. Apparently, proper fixation and processing of material is highly important for detection of LD wall components [9].

Recent reports suppose the active role of adipophilin in the transport of the lipids into the LDs from the cytosol [6]. Moreover mRNA of *PLIN2* seems to be overexpressed only during the active storage phase, which could be an argument for active transport role of this protein [6, 10, 11]. The primary site for LD biosynthesis is the endoplasmic reticulum [12]. Given the similarity of the LD wall, endoplasmic reticulum membrane and cell membrane, one could suppose that in certain circumstances adipophilin could participate in the formation of LDs directly at the cell membrane, which was to some extent confirmed in experimental works. Robenek et al. [12, 13] have found membrane expression of PLIN2 in macrophages. This expression was sparse under normal conditions but in case of extreme lipid load (lipid laden macrophages) expression was pronounced and clustered in elevated membrane domains corresponding to large LDs (and similar to those of endoplasmic reticulum). Authors consider the activation of LD formation machinery in cell membrane, while in case of lipid overload endoplasmic reticulum could be not able to cope with this task alone [12, 13]. This could be also confirmed in other studies [10, 14–16].

One of the typical features of the lipid droplets is their active efflux from the cell [17]. From the one side it makes possible the identification of PLIN2 and other wall components in the biological fluids, e.g. in RCC in urine [18] and in colon cancer in plasma [4]. From the other side, one could speculate that the lipid overload in clear cell tumors would certainly intensify the process of LD secretion through membrane. And this active transport would inevitably make the membrane immunopositive. The other putative cause for the membrane positivity is known as fusion of small lipid droplets [17] into one or several big containers within cytoplasm, which could relocate the LD wall to cell membrane imitating its positivity.

As for the functional role of adipophilin in tumor cells, the evidence is sparse. Although we and other groups have observed adipophilin accumulation in less aggressive CC-RCCs tumors, for some tumors accumulation of the lipid droplets seems to be a promoting event, e.g. in colon cancer [19] and breast cancer cell cultures [20], putatively giving the proliferative advantage to the cells and increasing their survival, which seems to be logical, - cells having more lipid droplets (and therefore more energy reserves and “building” material for new cells) could be more viable. Some other experimental studies provide a further support to this point of view, showing worse survival capabilities in cultured cells with impaired lipid droplet storage function (knockdown of adipophilin) [21]. In breast cancer cells adipophilin-rich cells also proliferated more actively (Ki-67 measured) [20]. This seems not to be a proper explanation of adipophilin overexpression in RCC.

REFERENCES

1. Oncomine database. http://www.oncomine.org.
2. Straub BK, Herpel E, Singer S, Zimbelmann R, Breuhahn K, Macher-Goeppinger S, Warth A, Lehmann-Koch J, Longerich T, Heid H, Schirmacher P. Lipid droplet-associated PAT-proteins show frequent and differential expression in neoplastic steatogenesis. Mod Pathol. 2010;23:480-92. doi: 10.1038/modpathol.2009.191
3. Rangel J, McCalmont TH. Intracytoplasmic adipophilin immunopositivity: a pitfall in the distinction of metastatic renal carcinoma from sebaceous carcinoma. J Cutan Pathol. 2010;37:1193-5. doi: 10.1111/j.1600-0560.2010.01635_2.x
4. Matsubara J, Honda K, Ono M, Sekine S, Tanaka Y, Kobayashi M, Jung G, Sakuma T, Nakamori S, Sata N, Nagai H, Ioka T, Okusaka T, Kosuge T, Tsuchida A, Shimahara M, Yasunami Y, Chiba T, Yamada T. Identification of adipophilin as a potential plasma biomarker for colorectal cancer using label-free quantitative mass spectrometry and protein microarray. Cancer Epidemiol Biomarkers Prev. 2011;20:2195-203. doi: 10.1158/1055-9965.EPI-11-0400
5. Najt CP, Lwande JS, McIntosh AL, Senthivinayagam S, Gupta S, Kuhn LA, Atshaves BP. Structural and functional assessment of perilipin 2 lipid binding domain(s). Biochemistry. 2014;53:7051-66. doi: 10.1021/bi500918m
6. McIntosh AL, Senthivinayagam S, Moon KC, Gupta S, Lwande JS, Murphy CC, Storey SM, Atshaves BP. Direct interaction of Plin2 with lipids on the surface of lipid droplets: a live cell FRET analysis. Am J Physiol Cell Physiol. 2012;303:C728-42. doi: 10.1152/ajpcell.00448.2011
7. Gross DN, Miyoshi H, Hosaka T, Zhang HH, Pino EC, Souza S, Obin M, Greenberg AS, Pilch PF. Dynamics of lipid droplet-associated proteins during hormonally stimulated lipolysis in engineered adipocytes: stabilization and lipid droplet binding of adipocyte differentiation-related protein/adipophilin. Mol Endocrinol 2006;20:459–66. doi: 10.1210/me.2005-0323#sthash.Keh4qcDE.dpuf
8. Takahashi Y, Shinoda A, Kamada H, Shimizu M, Inoue J, Sato R. Perilipin2 plays a positive role in adipocytes during lipolysis by escaping proteasomal degradation. Sci Rep. 2016;6:20975. doi: 10.1038/srep20975
9. Ohsaki Y, Maeda T, Fujimoto T. Fixation and permeabilization protocol is critical for the immunolabeling of lipid droplet proteins. Histochem Cell Biol. 2005;124:445-52.
10. Jiang HP, Serrero G. Isolation and characterization of a full-length cDNA coding for an adipose differentiation-related protein. Proc Natl Acad Sci U S A. 1992;89:7856-60.
11. Bickel PE, Tansey JT, Welte MA. PAT proteins, an ancient family of lipid droplet proteins that regulate cellular lipid stores. Biochim Biophys Acta. 2009 Jun;1791:419-40. doi: 10.1016/j.bbalip.2009.04.002
12. Robenek H, Buers I, Hofnagel O, Robenek MJ, Troyer D, Severs NJ. Compartmentalization of proteins in lipid droplet biogenesis. Biochim Biophys Acta. 2009;1791:408-18. doi: 10.1016/j.bbalip.2008.12.001
13. Robenek H, Robenek MJ, Buers I, Lorkowski S, Hofnagel O, Troyer D, Severs NJ. Lipid droplets gain PAT family proteins by interaction with specialized plasma membrane domains. J Biol Chem. 2005;280:26330-8. doi: 10.1074/jbc.M413312200
14. Ost A, Ortegren U, Gustavsson J, Nystrom FH, Strålfors P. Triacylglycerol is synthesized in a specific subclass of caveolae in primary adipocytes. J Biol Chem. 2005;280:5-8. doi: 10.1074/jbc.C400429200
15. Chong BM, Russell TD, Schaack J, Orlicky DJ, Reigan P, Ladinsky M, McManaman JL. The adipophilin C terminus is a self-folding membrane-binding domain that is important for milk lipid secretion. J Biol Chem. 2011;286:23254-65. doi: 10.1074/jbc.M110.217091
16. Gao J, Serrero G. Adipose differentiation related protein (ADRP) expressed in transfected COS-7 cells selectively stimulates long chain fatty acid uptake. J Biol Chem. 1999;274:16825-30. doi: 10.1074/jbc.274.24.16825
17. Boström P, Andersson L, Rutberg M, Perman J, Lidberg U, Johansson BR, Fernandez-Rodriguez J, Ericson J, Nilsson T, Borén J, Olofsson SO. SNARE proteins mediate fusion between cytosolic lipid droplets and are implicated in insulin sensitivity. Nat Cell Biol. 2007;9:1286-93. doi:10.1038/ncb1648
18. Morrissey JJ, Mellnick VM, Luo J, Siegel MJ, Figenshau RS, Bhayani S, Kharasch ED. Evaluation of Urine Aquaporin-1 and Perilipin-2 Concentrations as Biomarkers to Screen for Renal Cell Carcinoma: A Prospective Cohort Study. JAMA Oncol. 2015;1:204-12. doi: 10.1001/jamaoncol.2015.0213
19. Qi W, Fitchev PS, Cornwell ML, Greenberg J, Cabe M, Weber CR, Roy HK, Crawford SE, Savkovic SD. FOXO3 growth inhibition of colonic cells is dependent on intraepithelial lipid droplet density. J Biol Chem. 2013;288:16274-81. doi: 10.1074/jbc.M113.470617.
20. Pucer A, Brglez V, Payré C, Pungerčar J, Lambeau G, Petan T. Group X secreted phospholipase A(2) induces lipid droplet formation and prolongs breast cancer cell survival. Mol Cancer. 2013;12:111. doi: 10.1186/1476-4598-12-111
21. Bensaad K, Favaro E, Lewis CA, Peck B, Lord S, Collins JM, Pinnick KE, Wigfield S, Buffa FM, Li JL, Zhang Q, Wakelam MJ, Karpe F, Schulze A, Harris AL. Fatty acid uptake and lipid storage induced by HIF-1α contribute to cell growth and survival after hypoxia-reoxygenation. Cell Rep. 2014;9:349-65. doi: 10.1016/j.celrep.2014.08.056

Supplementary Table 1: Clinical characteristics of patients with clear-cell renal cell carcinoma (n=230)

Parameter	Absolute	Proportion
Age, years<br>mean (range)	61.1 (30-86)	-
Follow-up*, months<br>median (range)	100 (1-159)	-
Status to the end of follow-up		
alive	161	70.0%
dead	69	30.0%
Sex:		
male	150	65.2%
female	80	34.8%
pT-Stage (TNM 2002):		
pT1a	86	37.4%
pT1b	56	24.3%
pT2	13	5.7%
pT3a	25	10.9%
pT3b	45	19.6%
pT3c	3	1.3%
pT4	2	0.9%
WHO/ISUP grade:		
G1	46	20.0%
G2	133	57.8%
G3	35	15.2%
G4	16	7.0%
pN-Stage:		
N0	130	56.5%
N+	11	4.8%
Nx	89	38.7%
Lymphadenectomy		
Yes	129	56.1%
No	101	43.9%
M-Stage (clinical)		
M1 synchronic	15	6.5%
M1 asynchronic	11	4.8%
R-status		
R0	206	94.1%
R1	9	4.1%
R2	4	1.8%
not available	11	4.8%
Adrenalectomy		
Yes	149	64.8%
No	81	35.2%
ECOG performance status		
0	160	69.6%
1	64	27.8%
2	6	2.6%

Supplementary Table 2: Univariate analysis of prognostic significance of selected factors for overall survival in patients from the TCGA Cohort with clear cell renal cell carcinoma (including patients with G1)

N	Parameter	HR	95% Confidence interval	Significance
1	pT-stage			
	pT1	1	-	-
	pT2	1.44	0.82-2.52	0.202
	pT3a	3.61	2.45-5.33	9.68e-11
	pT3b	3.46	2.10-5.70	1.14e-06
	pT3c	8.21	1.98-34.13	0.004
	pT4	12.55	6.28-25.07	7.55e-13
2	pN-stage			
	pN0/Nx	1	-	-
	pN1	3.75	1.97-7.13	5.72e-05
3	WHO/ISUP grade*			
	G1	1	-	-
	G2	7.495e+06	0	Infinitive
	G3	1.412e+07	0	Infinitive
	G4	4.252e+07	0	Infinitive
4	WHO/ISUP grade (G1 excluded)			
	G2	1	-	-
	G3	1.88	1.26-2.81	0.002
	G4	5.67	3.72-8.66	8.88e-16
5	PLIN2 (Adipophilin) mRNA expression			
	High expression	1	-	-
	Low expression	1.76	1.28-2.42	0.0005

Comments: * - only 8 patients with G1 with uniform excellent overall survival.

Supplementary Table 3: Multivariate analysis of prognostic significance of selected factors for overall survival in patients from the TCGA Cohort with clear cell renal cell carcinoma (Patients with G1 excluded)

N	Parameter	HR	95% Confidence interval	Significance
1	pT-stage			
	pT1	1	-	-
	pT2	1.17	0.66-2.08	0.573
	pT3a	2.25	1.47-3.46	0.0002
	pT3b	2.45	1.45-4.13	0.0008
	pT3c	5.53	1.28-23.73	0.021
	pT4	3.56	1.43-8.86	0.006
2	pN-stage			
	pN0/Nx	1	-	-
	pN1	1.34	0.59-3.07	0.487
3	WHO/ISUP grade (G1 excluded)			
	G2	1	-	-
	G3	1.51	1.00-2.28	0.052
	G4	3.08	1.89-5.02	6.55e-06
4	PLIN2 (Adipophilin) mRNA expression			
	High expression	1	-	-
	Low expression	1.46	1.05-2.04	0.0257

Supplementay Table 4: Cut-off selection for PLIN2 (adipophilin) mRNA-expression using univariate Cox analysis for overall survival as endpoint

See Supplementary File 1
