# Supplementary material for: Adipophilin as prognostic biomarker in clear cell renal cell carcinoma
Source: Oncotarget. 2017 Feb 23;8(17):28672–82. doi: 10.18632/oncotarget.15639 (PMC5438682; doi:10.18632/oncotarget.15639)
Supplement: Supplementary file 2 [file oncotarget-08-28672-s002.docx]

**Suppl. Table 4**

Cut-off selection for PLIN2 (adipophilin) mRNA-expression using univariate Cox analysis for overall survival as endpoint

| Position | Cutoff | p-level |
| --- | --- | --- |
| 1 | 279.0383 | 1 |
| 2 | 296.9514 | 1 |
| 3 | 492.8511 | 1 |
| 4 | 551.3699 | 1 |
| 5 | 594.2934 | 1 |
| 6 | 614.4041 | 1 |
| 7 | 712.9048 | 1 |
| 8 | 744.7777 | 1 |
| 9 | 797.0286 | 1 |
| 10 | 855.9713 | 1 |
| 11 | 877.5928 | 1 |
| 12 | 894.9728 | 1 |
| 13 | 909.2845 | 1 |
| 14 | 945.7058 | 1 |
| 15 | 1043.6296 | 1 |
| 16 | 1203.1957 | 1 |
| 17 | 1393.5146 | 1 |
| 18 | 1405.7874 | 1 |
| 19 | 1408.2562 | 1 |
| 20 | 1491.5101 | 1 |
| 21 | 1580.5482 | 1 |
| 22 | 1708.6591 | 1 |
| 23 | 1723.0199 | 1 |
| 24 | 1832.8932 | 1 |
| 25 | 2152.7435 | 0.99999979 |
| 26 | 2197.066 | 0.99999981 |
| 27 | 2213.8614 | 0.99999981 |
| 28 | 2235.1589 | 0.99999997 |
| 29 | 2256.9775 | 0.99990575 |
| 30 | 2322.735 | 0.99878814 |
| 31 | 2367.4352 | 0.99983028 |
| 32 | 2445.0623 | 0.99999897 |
| 33 | 2506.9984 | 0.99993281 |
| 34 | 2597.7897 | 0.99996412 |
| 35 | 2652.3652 | 0.99999701 |
| 36 | 2692.7748 | 1 |
| 37 | 2852.4272 | 1 |
| 38 | 3018.0787 | 0.99999971 |
| 39 | 3107.4417 | 0.99999991 |
| 40 | 3248.3154 | 0.99993277 |
| 41 | 3375.1505 | 0.99995877 |
| 42 | 3427.5526 | 0.99876958 |
| 43 | 3560.9334 | 0.99118802 |
| 44 | 3693.1533 | 0.99236162 |
| 45 | 3720.6993 | 0.95881595 |
| 46 | 3744.5255 | 0.8814325 |
| 47 | 3783.0414 | 0.92126795 |
| 48 | 3879.056 | 0.95611642 |
| 49 | 4100.0063 | 0.9752152 |
| 50 | 4148.6762 | 0.91905051 |
| 51 | 4274.9731 | 0.95453088 |
| 52 | 4338.0192 | 0.97604609 |
| 53 | 4510.528 | 0.93055607 |
| 54 | 4621.9374 | 0.90312105 |
| 55 | 4636.0779 | 0.92022265 |
| 56 | 4798.5763 | 0.92593659 |
| 57 | 4855.0647 | 0.86704448 |
| 58 | 4868.7881 | 0.88202337 |
| 59 | 5216.6446 | 0.90897651 |
| 60 | 5277.8782 | 0.82140324 |
| 61 | 5590.1562 | 0.72421575 |
| 62 | 5669.145 | 0.62128737 |
| 63 | 5784.7255 | 0.66774274 |
| 64 | 5867.409 | 0.58344111 |
| 65 | 5877.5698 | 0.65064193 |
| 66 | 5893.5042 | 0.52541565 |
| 67 | 6037.8104 | 0.53396978 |
| 68 | 6125.8065 | 0.64407667 |
| 69 | 6301.4587 | 0.53977492 |
| 70 | 6328.1643 | 0.58446125 |
| 71 | 6346.5081 | 0.46497693 |
| 72 | 6416.6306 | 0.36191492 |
| 73 | 6441.9244 | 0.39425405 |
| 74 | 6490.8928 | 0.29291509 |
| 75 | 6523.6917 | 0.33059015 |
| 76 | 6562.4099 | 0.2524453 |
| 77 | 6639.2041 | 0.27439564 |
| 78 | 6660.9365 | 0.20862336 |
| 79 | 6675.5668 | 0.24907447 |
| 80 | 6680.6759 | 0.26468338 |
| 81 | 6710.3861 | 0.31266685 |
| 82 | 6818.4523 | 0.31902729 |
| 83 | 6956.6022 | 0.25241308 |
| 84 | 6999.1488 | 0.27925586 |
| 85 | 7063.9424 | 0.33284508 |
| 86 | 7086.977 | 0.38240517 |
| 87 | 7091.1074 | 0.2830248 |
| 88 | 7108.7191 | 0.3283299 |
| 89 | 7204.9129 | 0.26396884 |
| 90 | 7386.1947 | 0.36918557 |
| 91 | 7441.0632 | 0.41221186 |
| 92 | 7454.037 | 0.32661172 |
| 93 | 7576.1885 | 0.35866667 |
| 94 | 7581.4159 | 0.27166318 |
| 95 | 7729.9087 | 0.22284272 |
| 96 | 7744.3278 | 0.17724713 |
| 97 | 7805.0915 | 0.21064029 |
| 98 | 7838.9383 | 0.24040353 |
| 99 | 7864.8677 | 0.28868947 |
| 100 | 8007.835 | 0.2283266 |
| 101 | 8027.9813 | 0.25941278 |
| 102 | 8080.9595 | 0.31876267 |
| 103 | 8159.8911 | 0.35316782 |
| 104 | 8205.9553 | 0.36788853 |
| 105 | 8271.1938 | 0.45128015 |
| 106 | 8512.9405 | 0.45754698 |
| 107 | 8535.411 | 0.5066262 |
| 108 | 8613.9058 | 0.40458612 |
| 109 | 8628.1548 | 0.40600237 |
| 110 | 8641.9012 | 0.30357696 |
| 111 | 8647.8873 | 0.22052889 |
| 112 | 8715.4537 | 0.22813631 |
| 113 | 8782.646 | 0.19489642 |
| 114 | 8826.5345 | 0.22210696 |
| 115 | 8827.5218 | 0.15932731 |
| 116 | 8869.8067 | 0.16180967 |
| 117 | 8975.7013 | 0.18404024 |
| 118 | 9025.3725 | 0.13786011 |
| 119 | 9039.8371 | 0.15250692 |
| 120 | 9173.0475 | 0.11416737 |
| 121 | 9184.3375 | 0.13126813 |
| 122 | 9215.9032 | 0.14754358 |
| 123 | 9262.5438 | 0.11950335 |
| 124 | 9359.6838 | 0.0823252 |
| 125 | 9420.5878 | 0.0823252 |
| 126 | 9436.8824 | 0.05587736 |
| 127 | 9445.2808 | 0.04272733 |
| 128 | 9486.0692 | 0.02869714 |
| 129 | 9569.5117 | 0.02976676 |
| 130 | 9683.0654 | 0.03215741 |
| 131 | 9764.9214 | 0.03852164 |
| 132 | 9842.14 | 0.04036797 |
| 133 | 9864.2241 | 0.05032872 |
| 134 | 10134.099 | 0.03306478 |
| 135 | 10294.291 | 0.03741862 |
| 136 | 10376.6795 | 0.04675277 |
| 137 | 10431.3076 | 0.04916666 |
| 138 | 10455.6694 | 0.05150604 |
| 139 | 10478.5659 | 0.07149718 |
| 140 | 10804.9209 | 0.04878951 |
| 141 | 10808.5 | 0.03252924 |
| 142 | 10854.2464 | 0.02059094 |
| 143 | 10869.5951 | 0.02615203 |
| 144 | 10908.5277 | 0.0169805 |
| 145 | 10921.6224 | 0.02202918 |
| 146 | 10935.7755 | 0.02792855 |
| 147 | 11082.1686 | 0.03209586 |
| 148 | 11104.503 | 0.02554201 |
| 149 | 11118.5185 | 0.02857599 |
| 150 | 11152.9098 | 0.02028054 |
| 151 | 11175.9849 | 0.01441604 |
| 152 | 11357.6788 | 0.01914267 |
| 153 | 11424.3176 | 0.01965792 |
| 154 | 11534.9365 | 0.02241759 |
| 155 | 11582.4487 | 0.02840771 |
| 156 | 11600.6965 | 0.02993317 |
| 157 | 11810.8011 | 0.01975401 |
| 158 | 11826.7281 | 0.01580697 |
| 159 | 11965.9141 | 0.0114768 |
| 160 | 11974.5146 | 0.00805402 |
| 161 | 12007.2993 | 0.00978349 |
| 162 | 12057.5774 | 0.01266359 |
| 163 | 12077.7109 | 0.01290008 |
| 164 | 12171.9331 | 0.01491485 |
| 165 | 12211.3557 | 0.01978535 |
| 166 | 12212.6403 | 0.02195234 |
| 167 | 12284.466 | 0.02586634 |
| 168 | 12361.1219 | 0.02668775 |
| 169 | 12547.2945 | 0.01754031 |
| 170 | 12566.5658 | 0.02128541 |
| 171 | 12638.9245 | 0.02143687 |
| 172 | 12876.4585 | 0.02420139 |
| 173 | 13018.729 | 0.01576544 |
| 174 | 13081.262 | 0.01605335 |
| 175 | 13488.4218 | 0.02300636 |
| 176 | 13612.977 | 0.02913359 |
| 177 | 13621.8933 | 0.04793503 |
| 178 | 13661.6002 | 0.04987377 |
| 179 | 13831.4914 | 0.05885807 |
| 180 | 13909.2666 | 0.06935941 |
| 181 | 13975.5523 | 0.07642512 |
| 182 | 14029.9667 | 0.09347537 |
| 183 | 14040.1606 | 0.09902741 |
| 184 | 14083.7817 | 0.06917085 |
| 185 | 14100.2604 | 0.04746292 |
| 186 | 14139.1364 | 0.05124962 |
| 187 | 14146.4674 | 0.05302185 |
| 188 | 14152.1627 | 0.03448996 |
| 189 | 14156.5609 | 0.0397203 |
| 190 | 14229.2359 | 0.05111963 |
| 191 | 14369.5984 | 0.06609574 |
| 192 | 14489.5657 | 0.07786469 |
| 193 | 14737.9377 | 0.05571432 |
| 194 | 14794.8259 | 0.05697154 |
| 195 | 15116.0747 | 0.03812239 |
| 196 | 15130.1306 | 0.02436754 |
| 197 | 15184.4478 | 0.03080963 |
| 198 | 15188.9862 | 0.04248527 |
| 199 | 15261.9674 | 0.04356961 |
| 200 | 15267.2148 | 0.0344874 |
| 201 | 15299.0273 | 0.03539149 |
| 202 | 15315.8091 | 0.02743809 |
| 203 | 15325.8533 | 0.0275729 |
| 204 | 15385.0679 | 0.03242407 |
| 205 | 15462.0021 | 0.03485745 |
| 206 | 15508.6291 | 0.0320443 |
| 207 | 15611.4391 | 0.03373978 |
| 208 | 15657.0011 | 0.03844119 |
| 209 | 15665.5874 | 0.02593096 |
| 210 | 15731.9296 | 0.0210183 |
| 211 | 15755.6051 | 0.02759828 |
| 212 | 15836.6358 | 0.035313 |
| 213 | 16027.4299 | 0.02603362 |
| 214 | 16094.8859 | 0.01603962 |
| 215 | 16194.5269 | 0.01734649 |
| 216 | 16403.1564 | 0.02025252 |
| 217 | 16422.9767 | 0.02358473 |
| 218 | 16444.5792 | 0.01573579 |
| 219 | 16542.4893 | 0.00945113 |
| 220 | 16563.4855 | 0.00609695 |
| 221 | 16571.9782 | 0.00879218 |
| 222 | 16616.7076 | 0.01037662 |
| 223 | 16826.0352 | 0.01189248 |
| 224 | 16979.7978 | 0.01699141 |
| 225 | 17031.2733 | 0.01808262 |
| 226 | 17048.8586 | 0.02076289 |
| 227 | 17121.8659 | 0.02510769 |
| 228 | 17191.0934 | 0.01682502 |
| 229 | 17344.9105 | 0.01835078 |
| 230 | 17346.4494 | 0.02027925 |
| 231 | 17437.9179 | 0.02453498 |
| 232 | 17481.6564 | 0.02463242 |
| 233 | 17654.9794 | 0.01588213 |
| 234 | 17726.4438 | 0.01853017 |
| 235 | 17801.2987 | 0.01309835 |
| 236 | 17873.5245 | 0.00810762 |
| 237 | 17912.3377 | 0.00526482 |
| 238 | 17923.5105 | 0.00385283 |
| **239** | **17994.7796** | **0.00236206** |
| 240 | 17999.5044 | 0.00369681 |
| 241 | 18148.9755 | 0.00479008 |
| 242 | 18149.1195 | 0.00592311 |
| 243 | 18157.7607 | 0.00412319 |
| 244 | 18349.8026 | 0.00439174 |
| 245 | 18552.9995 | 0.00475793 |
| 246 | 18755.007 | 0.00552966 |
| 247 | 18758.4632 | 0.00355295 |
| 248 | 18947.5636 | 0.00415313 |
| 249 | 19055.2483 | 0.00428787 |
| 250 | 19099.3789 | 0.00445028 |
| 251 | 19182.5169 | 0.00528093 |
| 252 | 19334.72 | 0.00603231 |
| 253 | 19368.3822 | 0.00617554 |
| 254 | 19426.4151 | 0.00916257 |
| 255 | 19430.9411 | 0.00946327 |
| 256 | 19675.2137 | 0.01247134 |
| 257 | 19770.341 | 0.01318684 |
| 258 | 19869.6954 | 0.01334334 |
| 259 | 19967.4019 | 0.00804889 |
| 260 | 20145.4712 | 0.00886481 |
| 261 | 20177.7085 | 0.01046102 |
| 262 | 20252.1008 | 0.01066007 |
| 263 | 20291.2243 | 0.01463167 |
| 264 | 20336.7347 | 0.01956622 |
| 265 | 20407.4982 | 0.02369005 |
| 266 | 20440.1929 | 0.03261064 |
| 267 | 20451.7455 | 0.04544376 |
| 268 | 20653.4049 | 0.03197915 |
| 269 | 20680.9308 | 0.03820901 |
| 270 | 20735.7646 | 0.03024015 |
| 271 | 20810.2246 | 0.02660146 |
| 272 | 20849.8099 | 0.03355423 |
| 273 | 21272.6214 | 0.05473272 |
| 274 | 21297.684 | 0.06125597 |
| 275 | 21397.547 | 0.06274215 |
| 276 | 21421.2538 | 0.06484978 |
| 277 | 21741.4634 | 0.04417685 |
| 278 | 21766.0819 | 0.02941066 |
| 279 | 21848.8432 | 0.03209356 |
| 280 | 21875.581 | 0.02014862 |
| 281 | 22043.1951 | 0.01364175 |
| 282 | 22182.8921 | 0.00870795 |
| 283 | 22214.701 | 0.00884584 |
| 284 | 22318.502 | 0.0119107 |
| 285 | 22514.3224 | 0.01261461 |
| 286 | 22520.1631 | 0.01285028 |
| 287 | 22559.2946 | 0.01528674 |
| 288 | 22575.5906 | 0.01956658 |
| 289 | 22604.7393 | 0.02488453 |
| 290 | 22885.0972 | 0.01790328 |
| 291 | 22909.0674 | 0.03031691 |
| 292 | 23021.5866 | 0.01933623 |
| 293 | 23294.9274 | 0.01968176 |
| 294 | 23375.0486 | 0.02044793 |
| 295 | 23401.537 | 0.0274918 |
| 296 | 23673.7817 | 0.03178541 |
| 297 | 24023.1164 | 0.02345345 |
| 298 | 24062.0461 | 0.02409725 |
| 299 | 24064.5783 | 0.03150908 |
| 300 | 24109.883 | 0.03613849 |
| 301 | 24120.2823 | 0.044875 |
| 302 | 24223.4062 | 0.04909333 |
| 303 | 24364.0687 | 0.03228188 |
| 304 | 24608.6256 | 0.03292688 |
| 305 | 24648.5476 | 0.04322798 |
| 306 | 24677.7849 | 0.05622415 |
| 307 | 24725.2435 | 0.03615901 |
| 308 | 24974.0612 | 0.02714525 |
| 309 | 25041.7221 | 0.01719376 |
| 310 | 25479.4155 | 0.01177781 |
| 311 | 26096.1594 | 0.01436455 |
| 312 | 26098.9997 | 0.01731018 |
| 313 | 26161.5222 | 0.01836891 |
| 314 | 26248.2833 | 0.02288273 |
| 315 | 26363.2621 | 0.02653866 |
| 316 | 26379.038 | 0.01645496 |
| 317 | 26439.158 | 0.01918755 |
| 318 | 26516.1462 | 0.01327587 |
| 319 | 26562.5277 | 0.01620203 |
| 320 | 26604.8706 | 0.01358181 |
| 321 | 26662.0301 | 0.00823922 |
| 322 | 26681.3849 | 0.00871921 |
| 323 | 26753.3287 | 0.00654684 |
| 324 | 26773.4264 | 0.00969373 |
| 325 | 27331.5943 | 0.0078857 |
| 326 | 27380.7947 | 0.01182531 |
| 327 | 27384.6365 | 0.01360764 |
| 328 | 27561.7442 | 0.01776078 |
| 329 | 27704.9944 | 0.02444197 |
| 330 | 27710.1737 | 0.02945452 |
| 331 | 27796.0271 | 0.01997327 |
| 332 | 27841.7222 | 0.02237834 |
| 333 | 28162.443 | 0.02340767 |
| 334 | 28189.9547 | 0.03262294 |
| 335 | 28199.7972 | 0.04091857 |
| 336 | 28218.3607 | 0.04827684 |
| 337 | 28240.553 | 0.05254655 |
| 338 | 28244.9558 | 0.05913211 |
| 339 | 28254.6419 | 0.08032889 |
| 340 | 28368.7273 | 0.05925975 |
| 341 | 28422.9249 | 0.06214586 |
| 342 | 28542.4578 | 0.0660046 |
| 343 | 28556.238 | 0.05807186 |
| 344 | 28560.5398 | 0.03899068 |
| 345 | 28629.7611 | 0.03991395 |
| 346 | 28741.9488 | 0.04102764 |
| 347 | 28923.7668 | 0.05112735 |
| 348 | 28980.5422 | 0.05857305 |
| 349 | 28999.1005 | 0.06387222 |
| 350 | 29011.1082 | 0.0496829 |
| 351 | 29015.9262 | 0.05798925 |
| 352 | 29044.0355 | 0.05977172 |
| 353 | 29516.129 | 0.05922756 |
| 354 | 29835.6574 | 0.06540974 |
| 355 | 29841.8472 | 0.04469003 |
| 356 | 29931.3351 | 0.05287123 |
| 357 | 29989.4594 | 0.06539962 |
| 358 | 30034.696 | 0.09169911 |
| 359 | 30139.995 | 0.11463005 |
| 360 | 30289.3192 | 0.12315497 |
| 361 | 30370.2882 | 0.08979291 |
| 362 | 30535.4814 | 0.12383504 |
| 363 | 30560.4203 | 0.16242355 |
| 364 | 30860.3511 | 0.2126968 |
| 365 | 30987.3487 | 0.21840057 |
| 366 | 31054.9348 | 0.22675208 |
| 367 | 31989.4495 | 0.23620745 |
| 368 | 32197.5509 | 0.2675901 |
| 369 | 32512.6513 | 0.31272554 |
| 370 | 32595.4451 | 0.41673929 |
| 371 | 32661.7579 | 0.41940829 |
| 372 | 32728.0474 | 0.47907049 |
| 373 | 32747.9862 | 0.366414 |
| 374 | 32815.912 | 0.39225702 |
| 375 | 32904.5324 | 0.33213844 |
| 376 | 32949.2014 | 0.29326339 |
| 377 | 33115.8253 | 0.32266577 |
| 378 | 33164.3398 | 0.25911689 |
| 379 | 33233.8341 | 0.27545766 |
| 380 | 33305.9747 | 0.32150513 |
| 381 | 33367.1092 | 0.35323061 |
| 382 | 33386.817 | 0.27172853 |
| 383 | 33529.5406 | 0.32432889 |
| 384 | 33600.6787 | 0.36341112 |
| 385 | 33799.7481 | 0.42234943 |
| 386 | 34013.9475 | 0.46877461 |
| 387 | 34318.6359 | 0.47690713 |
| 388 | 34383.1543 | 0.54028184 |
| 389 | 34430.5452 | 0.45265163 |
| 390 | 34442.5641 | 0.51458971 |
| 391 | 34532.7841 | 0.57979993 |
| 392 | 34595.1826 | 0.62856461 |
| 393 | 35093.0771 | 0.66373086 |
| 394 | 35206.0616 | 0.66687056 |
| 395 | 35466.5592 | 0.59369348 |
| 396 | 35506.4856 | 0.67987052 |
| 397 | 35544.3038 | 0.71782305 |
| 398 | 36168.1548 | 0.62613601 |
| 399 | 36219.7266 | 0.66885834 |
| 400 | 36400.8733 | 0.66970101 |
| 401 | 36462.4338 | 0.71917479 |
| 402 | 36491.1189 | 0.78394968 |
| 403 | 36643.8632 | 0.73609985 |
| 404 | 37244.2712 | 0.64605303 |
| 405 | 37369.3931 | 0.67282156 |
| 406 | 37698.1062 | 0.81539419 |
| 407 | 37782.6892 | 0.82832077 |
| 408 | 38124.8224 | 0.720073 |
| 409 | 38263.7899 | 0.77174615 |
| 410 | 38494.3751 | 0.7774929 |
| 411 | 38863.7781 | 0.65024803 |
| 412 | 38895.1976 | 0.75353978 |
| 413 | 38956.3348 | 0.83729751 |
| 414 | 39154.2244 | 0.89012461 |
| 415 | 39208.1849 | 0.96925176 |
| 416 | 39208.8946 | 0.98659961 |
| 417 | 39268.6893 | 0.98902062 |
| 418 | 39775.2809 | 0.99408165 |
| 419 | 39876.013 | 0.99801209 |
| 420 | 40157.5592 | 0.99195735 |
| 421 | 40560.1469 | 0.99706087 |
| 422 | 40790.4745 | 0.99919214 |
| 423 | 40832.0463 | 0.99936556 |
| 424 | 40854.3619 | 0.99949276 |
| 425 | 41475.7452 | 0.99983919 |
| 426 | 41532.5353 | 0.99999792 |
| 427 | 41774.4385 | 1 |
| 428 | 42270.1422 | 1 |
| 429 | 42558.5369 | 1 |
| 430 | 42701.1778 | 0.99999999 |
| 431 | 42833.7939 | 1 |
| 432 | 43165.8067 | 1 |
| 433 | 43400.645 | 1 |
| 434 | 43702.7276 | 1 |
| 435 | 43778.8945 | 1 |
| 436 | 44082.4445 | 0.99999974 |
| 437 | 44382.7801 | 1 |
| 438 | 45312.7542 | 0.99999744 |
| 439 | 45721.393 | 0.99993221 |
| 440 | 45845.1919 | 0.9999999 |
| 441 | 46194.3646 | 0.99985285 |
| 442 | 46645.732 | 0.99991711 |
| 443 | 47193.7221 | 0.99995492 |
| 444 | 47605.087 | 0.99999683 |
| 445 | 47929.3893 | 0.99999993 |
| 446 | 47952.7311 | 1 |
| 447 | 48707.0871 | 0.99999302 |
| 448 | 48894.9809 | 0.99939391 |
| 449 | 49118.5609 | 0.99975043 |
| 450 | 49314.8961 | 0.99999991 |
| 451 | 49541.5385 | 1 |
| 452 | 50454.2396 | 1 |
| 453 | 50574.923 | 1 |
| 454 | 50642.4427 | 1 |
| 455 | 50680 | 0.99999953 |
| 456 | 50953.4788 | 0.99996201 |
| 457 | 51098.3924 | 0.9999975 |
| 458 | 51872.4247 | 0.99999995 |
| 459 | 52007.7875 | 0.99999998 |
| 460 | 52106.9824 | 1 |
| 461 | 53573.7941 | 1 |
| 462 | 53825.5275 | 1 |
| 463 | 53841.9865 | 1 |
| 464 | 54167.1985 | 1 |
| 465 | 56353.687 | 1 |
| 466 | 56761.745 | 1 |
| 467 | 57955.3073 | 1 |
| 468 | 58046.7009 | 1 |
| 469 | 58966.5394 | 1 |
| 470 | 59470.392 | 1 |
| 471 | 60659.3592 | 1 |
| 472 | 61109.6582 | 1 |
| 473 | 61805.5866 | 1 |
| 474 | 62209.9395 | 1 |
| 475 | 62473.3248 | 1 |
| 476 | 62812.5408 | 1 |
| 477 | 62883.8618 | 1 |
| 478 | 63237.7688 | 1 |
| 479 | 63704.7069 | 1 |
| 480 | 68250.6351 | 1 |
| 481 | 68607.4328 | 1 |
| 482 | 69078.0471 | 1 |
| 483 | 69563.2527 | 1 |
| 484 | 70333.7976 | 1 |
| 485 | 75576.4936 | 1 |
| 486 | 84475.3162 | 1 |
| 487 | 103143.445 | 1 |
| 488 | 105465.14 | 1 |
| 489 | 159406.957 | 1 |
